# Supplementary material for: Genetic Enhancement for Biotic Stress Resistance in Basmati Rice through Marker-Assisted Backcross Breeding
Source: Int J Mol Sci. 2023 Nov 8;24(22):16081. doi: 10.3390/ijms242216081 (PMC10671030; doi:10.3390/ijms242216081)
Supplement: Supplementary file 1 [file ijms-24-16081-s001.zip › ijms-2548457-supplementary.pdf]

International Journal of Molecular Sciences

MDPI

Supplementary Material

Genetic Enhancement for Biotic Stress Resistance in Basmati Rice through Marker-Assisted Backcross Breeding

Gagandeep Singh [1,†], Niraj Singh [1,†], Ranjith Kumar Ellur [1], Alexander Balamurugan [2], G. Prakash [2], Rajeev Rathour [3], Kalyan Kumar Mondal [2], Prolay Kumar Bhowmick [1], S. Gopala Krishnan [1], Mariappan Nagarajan [4], Rakesh Seth [5], K. K. Vinod [1], Varsha Singh [1], Haritha Bollinedi [1] and Ashok Kumar Singh [1,*]

1 Division of Genetics, ICAR-Indian Agricultural Research Institute, New Delhi 110012, India; gagan.singh269@gmail.com (G.S.); niraj.singh37@gmail.com (N.S.); ranjith@iari.res.in or ranjithellur@gmail.com (R.K.E.); prolaybhowmick@gmail.com (P.K.B.); gopal_icar@yahoo.co.in (S.G.K.); kkvinodh@gmail.com (K.K.V.); varshagpb.10aug@gmail.com (V.S.); haritha.agrico@gmail.com (H.B.)
2 Division of Plant Pathology, ICAR-Indian Agricultural Research Institute, New Delhi 110012, India; abalamurugan555@gmail.com (A.B.); prakashg.ganesan@gmail.com (G.P.); mondaliari@gmail.com (K.K.M.)
3 Department of Agriculture Biotechnology, CSKHPKV, Palampur 176062, Himachal Pradesh, India; rathour72@gmail.com
4 Rice Breeding and Genetics Research Centre, ICAR-Indian Agricultural Research Institute, Aduthurai 612101, Tamil Nadu, India; iariadt@gmail.com
5 Regional Station, ICAR-Indian Agricultural Research Institute, Karnal 132001, Haryana, India; rseth101@gmail.com
* Correspondence: aksingh@iari.res.in
† These authors contributed equally to this work.

Supplementary table S1. List of polymorphic markers between PB1509 and Pusa Basmati 1718, their location and the amplified fragment size

Marker	Position	Location (Mb)	Amplicon size (bp)	
			PB1509	PB1718
RM 1344	1	7.02	170	150
RM 35	1	8.41	240	250
RM 572	1	9.86	170	150
RM 10854	1	14.04	400	700
RM 5919	1	24.74	330	300
RM 1152	1	30.09	280	270
RM 11983	1	38.48	110	90

RM 431	1	38.89	245	235
RM 104	1	40.16	240	210
RM 1067	1	42.91	240	310
RM 12273	1	42.99	300	320
RM 236	2	2.11	300	250
HVSSR2-10	2	4.29	280	300
RM 3732	2	4.4	150	140
RM 6247	2	5.79	100	120
RM 12718	2	6.5	200	250
RM 12808	2	7.74	450	430
RM 12811	2	7.77	200	210
RM 12812	2	7.77	240	315
RM 71	2	8.76	160	130
RM 7144	2	8.81	100	130
RM 452	2	9.56	270	250
RM 13088	2	13.06	350	340
RM 13114	2	13.95	240	220
RM 13181	2	16.06	160	150
RM 13217	2	16.91	320	300
RM 13254	2	17.86	500	530
RM 13316	2	19.05	250	280
RM 1379	2	20.05	380	350
RM 262	2	20.79	170	150
RM 263	2	25.88	120	100
RM 13672	2	26	140	130
RM 5404	2	33.7	200	220
RM 5460	2	33.79	130	150
RM 208	2	35.16	180	160
RM 561	2	35.93	160	180
RM 175	3	3.84	200	190
OSR 13	3	7.12	60	80
RM 3646	3	21.79	180	200
RM 15857	3	29.67	120	100
RM 335	4	0.68	110	160
RM 401	4	13.15	280	240
RM 317	4	29.06	200	220
RM 7293	5	7.42	150	140
RM 169	5	7.49	170	200
RM 18297	5	12.48	150	160
RM 217	6	31.24	200	150
RM 51	7	0.24	150	120
RM 21311	7	8.69	680	700
RM 336	7	21.81	240	210
RM 455	7	22.35	80	165
RM 447	8	26.54	80	120
RGNMS 2921	8	29.82	200	215
RM 6100	10	18.37	150	140
RM 1108	10	18.71	120	110
RM 147	10	21.02	80	100

RM 590	10	23.11	135	125
RM 26777	11	17.32	200	210
RM 457	11	18.86	250	240
RM 7443	11	28.23	135	145
RM 20A	12	0.97	110	125
RM 7619	12	4.83	185	150
RM 27841	12	8.02	250	280
RM 465	12	16.8	200	190
RM 511	12	17.44	150	140
RM 1261	12	17.57	130	160
RM 277	12	18.32	140	130
RM 1246	12	19.16	130	160
RM 28277	12	19.45	260	250
RM 519	12	19.97	140	110
RM 463	12	22.16	200	210
RM 3226	12	24.07	180	200
RM 235	12	26.17	200	190

Supplementary table S2. List of polymorphic markers between PB1509 and P1927, their location and the amplified fragment size

**Marker**	**Position**	**Location (Mb)**	**Amplicon size (bp)**	
			**PB1509**	**P1927**
RM 1344	1	7.02	170	150
RM 35	1	8.41	240	250
RM 572	1	9.86	170	150
RM 24	1	18.93	150	170
RM 11365	1	25	280	270
RM 15857	1	27.62	120	100
RM 11983	1	38.48	110	90
RM 431	1	38.89	245	235
RM 3482	1	39.71	150	200
RM 104	1	40.16	240	210
RM 12273	1	42.99	300	320
HVSSR 2-1	2	0.12	300	280
RM 154	2	1.08	200	180
HVSSR2-10	2	4.29	280	300
RM 6247	2	5.79	100	120
RM 12811	2	7.77	200	210
RM 7144	2	8.81	100	130
RM 237	2	9.56	100	80
RM 12718	2	13.06	200	250
RM 13088	2	13.95	350	340
RM 13114	2	14.92	240	220

RM 13181	2	15.96	160	150
RM 13217	2	17.86	320	300
RM 13263	2	18.08	100	120
RM 13254	2	19.05	500	530
RM 262	2	20.27	170	150
HVSSR 2-59	2	22.14	200	150
RM 3275	2	22.45	190	240
RM 263	2	25.86	120	100
RM 1379	2	26	380	350
RM 3730	2	30.1	140	160
RM 7443	2	32.02	135	145
RM 5404	2	33.67	200	220
RM 5460	2	33.76	130	150
RM 561	2	34.42	160	180
RM 208	2	35.13	180	160
RM 71	3	1.52	160	130
RM 452	3	4.33	270	250
OSR 13	3	7.12	60	80
RM 13672	3	14.19	140	130
RM 5919	3	29.67	330	300
RM 335	4	0.68	110	140
RM 1261	4	3.12	130	170
RM 169	4	13.15	170	200
RM 18297	4	16.63	150	160
RM 175	5	7.49	200	190
RM 7619	5	12.48	185	150
RM 20A	5	20.9	110	125
RM 217	6	4.18	200	150
RM 317	6	17.57	200	220
RM 51	7	0.34	150	120
RM 21311	7	17.82	680	700
RM 336	7	21.81	240	210
RM 455	7	22.35	80	165
RM 6022	7	29.39	100	110
RM 12812	8	27.9	240	315
RM 6100	10	0.31	150	140
RM 457	10	2.62	250	240
RM 6271	10	4.43	100	80
RM 271	10	16.2	80	100
RM 1108	10	18.71	120	110
RM 147	10	20.94	80	100
RM 3226	11	19.43	180	220
RM 21	11	25.15	150	170
RM 7110	11	26.45	190	200
RM 28277	12	0.43	260	250
RM 590	12	4.83	135	125
RM 12692	12	7.43	200	190
RM 465	12	12.83	200	190
RM 511	12	17.39	150	140

RM 1246	12	19.15	130	160
RM 519	12	19.9	140	110
RM 28326	12	20.24	110	130
RM 277	12	21.16	140	130
HVSSR 12-36	12	21.16	300	280
RM 463	12	22.15	200	210
RM 235	12	26.17	200	190

Supplementary table S3. List of polymorphic markers between PB1509 and P1929, their location and the amplified fragment size

Marker	Position	Location (Mb)	Amplicon size (bp)	
			PB1509	P1929
RM 495	1	0.21	225	210
RM 499	1	0.38	80	90
RM 246	1	27.33	130	120
RM 1232	1	27.62	130	120
RM 1152	1	30.09	270	260
RM 11738	1	32.92	220	320
RM 212	1	33.05	130	180
RM 1067	1	42.91	220	260
RM 237	1	43.27	140	130
RM 6247	2	5.79	100	120
RM 12692	2	6.09	180	160
RM 12811	2	7.77	270	520
RM 71	2	8.76	140	120
RM 7144	2	8.81	90	120
RM 452	2	9.56	210	200
RM 1178	2	14.01	325	250
HV 2-45	2	17.08	200	210
RGNMS 2921	2	19.65	190	210
RM 262	2	20.8	160	150
RM 263	2	25.88	120	100
RM 13672	2	26	180	170
HV 2-85	2	35.36	100	130
RM 138	2	35.68	235	225
RM 6139	2	35.83	325	310
RM 3872	3	6.82	125	135
RM 15002	3	14.19	560	570
RM 3646	3	21.79	140	150
RM 16	3	23.12	180	200
RM 15613	3	25.77	290	300
RM 520	3	30.92	260	250
RM 3867	3	31.75	95	110
RM 1352	3	32.34	235	210
RM 55	3	36.41	220	235

RM 16450	4	4.99	400	215
RM 16611	4	11.7	90	80
RM 307	4	13.15	190	130
RM 16745	4	16.63	190	180
RM 317	4	29.06	150	160
RM 19260	6	0.57	590	290
RM 8060	6	0.72	270	300
RM 19268	6	0.81	335	350
RM 19285	6	1.09	210	190
RM 19301	6	1.35	110	120
RM 1369	6	1.5	270	230
RM 1261	6	17.57	300	290
RM 162	6	24.04	230	250
RM 217	6	31.24	300	290
RM 6050	7	22.97	180	160
RM 1335	7	28.24	110	120
RM 152	8	0.68	160	150
RM 1235	8	1.21	130	140
RM 25	8	4.38	130	150
RM 316	9	1.02	200	210
RM 444	9	5.92	190	150
RM 23899	9	6.94	200	240
RM 6920	9	6.95	180	200
RM 23928	9	7.52	310	230
RM 23982	9	8.54	160	200
RM 257	9	17.71	150	200
RM 288	9	18.56	130	120
RM 160	9	19.75	130	100
RM 205	9	22.72	130	150
RM 222	10	2.62	200	190
RM 6100	10	10.89	145	130
RM 467	10	13.49	190	200
RM 147	10	21.02	110	120
RM 590	10	23.11	160	150
RM 3185	11	9.12	135	140
RM 26499	11	10.9	125	110
RM 26621	11	14.36	260	350
RM 7226	11	14.51	185	170
RM 287	11	17.23	120	110
RM 1341	11	19.43	150	170
RM 415	12	0.43	215	200
RM 20A	12	0.97	160	180
RM 247	12	3.19	170	130
RM 28032	12	13.64	250	260
RM 28059	12	14.53	380	390
HVSSR 12-36	12	21.16	380	330
RGNMS 3781	12	21.23	180	210
RM 235	12	26.17	130	110
RM 19	12	26.6	220	250

Supplementary table S4. List of polymorphic markers between PB1509 and Tetep, their location and the amplified fragment size

Marker	Position	Location (Mb)	Amplicon size (bp)	
			PB1509	Tetep
RM 499	1	0.38	80	90
RM 22	1	1.5	140	150
RM 8068	1	1.66	210	270
RM 84	1	11.56	130	150
RM 140	1	12.3	310	325
RM 24	1	18.98	160	180
RM 5919	1	24.73	280	270
RM 246	1	27.33	130	100
RM 1152	1	30.09	270	260
RM 11738	1	32.92	220	250
RM 12204	1	41.8	160	180
RM 1067	1	42.91	220	260
RM 154	2	1.08	180	165
RM 12415	2	1.76	280	290
RM 6247	2	5.79	100	120
RM 12811	2	7.77	270	520
RM 71	2	8.76	140	120
RM 7144	2	8.81	240	290
RM 5699	2	8.98	240	260
RM 1178	2	14.01	325	250
HV 2-45	2	17.08	200	210
RGNMS 2921	2	19.65	190	210
RM 13672	2	26	180	160
RM 3730	2	26.26	150	170
RM 221	2	27.62	135	150
HV 2-73	2	28.2	200	190
RM 525	2	28.26	140	100
RM 3275	2	30.1	165	180
RM 112	2	32.02	90	80
RM 208	2	35.16	180	160
HV 2-85	2	35.36	100	130
RM 138	2	35.68	235	225
OSR13	3	0.79	95	135
RM 3872	3	6.82	125	135
RM 157A	3	9.46	130	140
RM 15002	3	14.19	560	570
RM 16	3	23.12	180	200
RM 168	3	27.89	150	140
RM 520	3	30.92	260	250
RM 3867	3	31.75	95	110

RM 1352	3	32.34	235	250
RM 514	3	35.07	260	230
RM 570	3	35.38	250	260
HVSSR3-93	3	36	240	220
RM 55	3	36.41	220	235
RM 16294	4	0.92	350	280
RM 7200	4	4.03	215	200
RM 16450	4	4.99	400	340
RM 3471	4	6.31	140	160
RM 307	4	13.15	190	130
HV4-28	4	21.28	370	350
RM 3288	4	27.35	190	200
RM 2441	4	27.86	150	160
RM 317	4	29.06	150	160
RM 3687	4	31.29	170	160
RM 17512	4	31.83	285	275
HVSSR04-49	4	33.27	380	350
RM 131	4	34.61	160	150
RM 507	5	0.07	200	180
RM 6271	5	4.43	85	75
RM 169	5	7.49	160	190
RM 581	5	9.1	135	150
RM 18297	5	12.48	110	120
RM 146	5	17.96	350	360
RM 31	5	28.61	160	145
RM 19260	6	0.57	590	290
RM 8060	6	0.72	270	300
RM 19268	6	0.81	335	350
RM 1369	6	1.5	270	260
RM 111	6	5.09	280	270
RM 136	6	8.75	65	75
RM 1261	6	17.57	170	160
RM 275	6	24.32	250	260
RM 217	6	31.24	150	160
RM 8035	7	14.06	95	110
RM 2	7	16.02	170	160
RM 7110	7	16.8	190	200
RM 11	7	19.25	130	150
RM 6050	7	22.97	140	155
RM 234	7	25.42	140	130
RM 420	7	29.39	190	200
RM 25	8	4.38	130	150
RM 44	8	11.76	110	100
RM 3281	8	13.36	140	150
RM 23073	8	19.57	160	380
RM 284	8	21.13	150	135
RM 23741	9	2.68	400	410
RM 23899	9	6.94	200	240
RM 6920	9	6.95	140	150

RGNMS3037	9	7.2	300	310
RM 23928	9	7.52	310	280
RM 105	9	12.55	125	135
RM 24181	9	12.56	115	100
RM 24183	9	12.6	330	320
HV09-25	9	14.74	380	360
RM 3700	9	15.42	150	140
HV9-36	9	16.81	310	335
RM 160	9	19.75	130	100
RM 201	9	20.17	150	165
RGNMS3253	10	0.31	330	360
RM 271	10	4.9	110	120
RM 6100	10	10.89	145	130
RM 1375	10	16.2	160	180
RGNMS3297	10	16.26	310	290
RM 184	10	16.35	210	200
RM 1108	10	18.71	135	125
RM 171	10	19.04	350	340
RM 1108	10	19.23	125	115
RM 147	10	21.02	110	120
RM 1812	11	2.41	135	155
RM 332	11	2.84	160	150
RM 552	11	4.85	170	180
RM 26323	11	7.37	160	175
RM 536	11	8.89	210	230
RM 26499	11	10.9	140	125
RM 26544	11	11.8	170	160
RM 3428	11	13.48	150	160
RM 26621	11	14.36	260	350
RM 7226	11	14.51	180	190
RM 2110	11	19.22	150	110
RM 1341	11	19.43	150	170
RM 4601	11	22.25	120	170
RM 7443	11	28.23	130	120
RM 415	12	0.43	215	200
RM 20A	12	0.97	160	180
RM 247	12	3.19	170	130
RM 491	12	3.58	250	360
RM 28032	12	13.64	250	260
RM 1261	12	17.57	180	170
RM 6022	12	19.95	100	90
HVSSR 12-36	12	21.16	380	330
RGNMS 3781	12	21.23	180	210
RM 235	12	26.17	130	140
RM 19	12	26.6	220	250

Supplementary table S5. Blight resistance evaluation of the developed NILs with *Xoo* Race 4

NIL	Genotype	Genes	Lesion length (in cm)
G11	Pusa 3037-1-44-3-164-20-249-2	*xa13+Xa21*	3.49±0.80
G12	Pusa 3037-1-44-3-164-21-255-1	*xa13+Xa21*	2.04± 0.38
G13	Pusa 3037-1-44-3-164-21-256-4	*xa13+Xa21*	2.85± 0.52
G14	Pusa 3037-1-45-5-165-22-259-4	*xa13+Xa21*	1.68± 0.44
G15	Pusa 3054-2-47-7-166-24-261-1	*Xa38*	0.45± 0.13
G16	Pusa 3054-2-47-7-166-24-261-2	*Xa38*	2.56 ±0.61
G17	Pusa 3054-2-47-7-166-24-261-3	*Xa38*	2.76 ±0.80
G18	Pusa 3054-2-47-7-166-24-262-2	*Xa38*	0.33± 0.06
G19	Pusa 3054-2-47-7-166-24-262-3	*Xa38*	1.12± 0.47
G20	Pusa 3122-27-15-165-2	*xa13+Xa21+Xa38*	0.49± 0.11
G21	Pusa 3122-27-16-166-1	*xa13+Xa21+Xa38*	0.25±0.05
G22	Pusa 3124-37-17-168-4	*Xa38+Pi9+Pib*	0.38± 0.04
G23	Pusa 3124-37-18-175-1	*Xa38+Pi9+Pib*	0.36± 0.08
G24	Pusa 3124-38-19-176-5	*Xa38+Pi9+Pib*	0.23± 0.01
G25	Pusa 3124-40-21-179-4	*Xa38+Pi9+Pib*	0.21± 0.00
G26	Pusa 3124-40-22-180-2	*Xa38+Pi9+Pib*	0.43±0.09
-	PB1509	-	14.37± 0.71
-	PB1718	*xa13+Xa21*	1.77± 0.45
-	P1927	*Xa38*	0.61± 0.17
